# The Aversion Function of the Limbic Dopaminergic Neurons and Their Roles in Functional Neurological Disorders

**DOI:** 10.3389/fcell.2021.713762

**Published:** 2021-09-20

**Authors:** Zhengming He, Yao Jiang, Simeng Gu, Dandan Wu, Duo Qin, Guangkui Feng, Xianjun Ma, Jason H. Huang, Fushun Wang

**Affiliations:** ^1^Institute of Brain and Psychological Sciences, Sichuan Normal University, Chengdu, China; ^2^Department of Psychology, Jiangsu University Medical School, Zhenjiang, China; ^3^Children’s Hospital of Nanjing Medical University, Nanjing, China; ^4^School of Foreign Languages, China University of Geosciences, Wuhan, China; ^5^Department of Neurology, Lianyungang Hospital of Chinese Medicine, Affiliated Hospital of Nanjing University of Chinese Medicine, Nanjing, China; ^6^Department of Surgery, Texas A&M University College of Medicine, Temple, TX, United States

**Keywords:** limbic system, DA, aversion function, prediction error, functional neurological disorders

## Abstract

The Freudian theory of conversion suggested that the major symptoms of functional neurological disorders (FNDs) are due to internal conflicts at motivation, especially at the sex drive or libido. FND patients might behave properly at rewarding situations, but they do not know how to behave at aversive situations. Sex drive is the major source of dopamine (DA) release in the limbic area; however, the neural mechanism involved in FND is not clear. Dopaminergic (DAergic) neurons have been shown to play a key role in processing motivation-related information. Recently, DAergic neurons are found to be involved in reward-related prediction error, as well as the prediction of aversive information. Therefore, it is suggested that DA might change the rewarding reactions to aversive reactions at internal conflicts of FND. So DAergic neurons in the limbic areas might induce two major motivational functions: reward and aversion at internal conflicts. This article reviewed the recent advances on studies about DAergic neurons involved in aversive stimulus processing at internal conflicts and summarizes several neural pathways, including four limbic system brain regions, which are involved in the processing of aversion. Then the article discussed the vital function of these neural circuits in addictive behavior, depression treatment, and FNDs. In all, this review provided a prospect for future research on the aversion function of limbic system DA neurons and the therapy of FNDs.

## Introduction

Functional neurological disorders (FNDs) are a common mental disorder (Ludwig et al., 2018). FND is a medical condition in which there is a problem with the functioning of the brain, rather than a structural disease. The exact prevalence of FND is unknown; research suggests that FND is the second most common reason for a neurological outpatient visit after headache/migraine, accounting for one-sixth of diagnoses (Voon et al., 2016). This means that FND is as common as multiple sclerosis or Parkinson’s disease (PD). FND was firstly known as conversion disorder, which was introduced by Breuer and Freud (1895–1995), who suggested that FNDs are due to the contradiction in the affective idea, which is converted into a somatic phenomenon. The Freudian theory of conversion proposed that the major symptoms of FND are due to inhibition of internal motivation, especially the sex drive or libido. FND patients always try to hide their internal motivations and always behave like acting in a play. FND patients might behave properly at happy situations; but they do not behave properly at aversive situations, when they are trying to hide their emotions. Sex drive is the major source of dopamine (DA) release in the brain; however, how DA in the limbic area is involved in FND is not clear. DA has been known as a reward neurotransmitter in the brain, and a large number of studies have shown that the secretion of DA in the limbic area has an inseparable relationship with joy (Gu et al., 2015). Since the 1960s, a great number of studies have confirmed the vital role of DA in the process of reward and motivation (Fouriezos and Wise, 1976; Corbett and Wise, 1980), so DA has gradually become a synonym for joy (Wang F. et al., 2020). However, what happens to the DA at the internal conflict for FND patients?

Schultz (Schultz et al., 1997) and others put forward the viewpoint of reward prediction error for DA in the 1990s, which induced most of the studies about dopaminergic (DAergic) neurons focusing on reward prediction error in the following 20 years. Meanwhile, there are an increasing number of studies investigating the neural circuitry of reward prediction error during this period. Brain regions such as the ventral tegmental area (VTA), substantia nigra (SN) pars compacta (SNc) (He et al., 2021), nucleus accumbens (NAc), striatum, lateral hypothalamus (LHa), and lateral habenula (LHb) have been found to be critically associated with the prediction error for rewards (Bromberg-Martin et al., 2010; Morales and Margolis, 2017). The most interesting thing is that the DAergic neurons are not only involved in reward-related prediction error, but they are also in the prediction of aversive information (Matsumoto and Hikosaka, 2009). Therefore, DA might work for both rewarding and aversive predictions, and DAergic neurons might induce two main motivational functions: reward and aversion at internal conflicts (Bromberg-Martin et al., 2010).

There have been many reports about the role of DA in reward, so this article only discusses the aversive function of DAergic neurons and their neural circuits. First, we briefly explain the role of DA neurons in the limbic system and the possible aversion mechanisms for FND. Then we review the neural circuits that limbic system DA neurons participate in. Finally, in order to better understand the neural mechanisms of aversion, we summarize the shortcoming of current research and recommend future studies, to provide new ideas for neuropsychiatric diseases, such as addiction, depression, and FND.

## Aversion Function of Dopamine

Historically, FND has traditionally been viewed as an entirely psychological disorder in which repressed psychological stress or trauma gets “converted” into a physical symptom. This is where the term “conversion disorder” comes from. Psychological disorders and stressful life events, both recent and in childhood, may be risk factors for developing the condition in some patients, but they rarely provide a full explanation for the cause of the condition and are absent in many patients. Modern theories propose that FND has many causes, especially the monoamine neurotransmitters (Liang et al., 2021).

Dopaminergic neurons synthesize and release DA to a large area of the brain and change the emotional states of the whole brain (Morales and Margolis, 2017), including positive and negative reinforcement, decision making, and motivation (Adcock et al., 2006; Brischoux et al., 2009). It is revealed that DA is vital to the establishment of memory relevant to the search for reward motivation and reward cues, according to Dalley et al. (2005), and for the maintenance and promotion of action (i.e., motivation). However, the function of DA motivation was thought to be limited to approaching motivation or rewarding behavior. With the technological advancement, such as microdialysis methods, voltammetry method, and other electrophysiological methods, further studies (Salamone and Correa, 2012) found that DAergic neurons are also found to be involved non-reward events (Pezze and Feldon, 2004; Lisman and Grace, 2005; Redgrave and Gurney, 2006); for example, some researchers believe that DAergic neurons are also involved in the processing of other non-reward signals related to surprise, novelty, specificity, and even aversion. Surprisingly, DA has been shown to be involved in the opposite processes of reward, the aversion, which is also crucial for individual’s adaptation to environmental changes and cognitive processes such as learning and memory (Pignatelli and Bonci, 2015).

The pursuit of reward and the avoidance of punishment are two fundamental forces that drive animal’s behavior (Hu, 2016). Both Pavlov’s classical conditioned reflex and Skinner’s operational conditioned reflex have suggested that the pursuit of reward and the avoidance of punishment is beneficial evolutionally (Roy et al., 2014). Reward invokes approaching behaviors and induces satisfaction, which ultimately leads to behavior reinforcement, while an aversive stimulus produces negative emotions, including dislike and fear, resulting in avoidance and reducing the production of similar behaviors (Salamone and Correa, 2012; Hu, 2016).

## Aversion

Reward and aversion are two major important components for motivational control (Bromberg-Martin et al., 2010). DAergic neurons in the limbic system have been shown to be involved in these processes and cooperate with different neural circuits to coordinate the downstream cognitive structure and to control the motivational behavior (Bromberg-Martin et al., 2010; Fagan et al., 2020). The term “motivation” is widely used for reward (Salamone and Correa, 2012), and it is defined by the famous German philosopher Schopenhauer (1999) as “choose, seize, and even seek out the means of satisfaction.” Early psychologists such as Wundt, James, and Freud have all talked about motivation. However, motivation also involves avoiding, so a broad definition of motivation is the mental process, or an inner psychological process that is involved in approaching or avoiding a target. Young (1961) defined motivation as “the process of arousing actions, sustaining the activity in progress, and regulating the pattern of activity.” Peng (2012) defined motivation as an inner psychological process or internal motivation, guided, inspired, and maintained by a goal or object. The latest definition of motivation by Salamone (1992) is a series of processes by which organisms regulate the possibility, proximity, and availability of stimuli. Due to the phased characteristics of motivation, Salamone (2010) further defined stages of motivation as “desire, preparation, action, approach or seek.” FND patients might show some abnormal behaviors when the motivation was inhibited.

Given that motivation is a psychological process that consists of a series of phased psychological processes, including reward and aversion, reward motivation as approaching or enjoying an object or event induces positive emotion or pleasure (Schultz, 2010). According to Berridge and Kringelbach (2015), a similar definition of reward is that rather than a single process, reward contains several psychological components, namely, liking, wanting, and learning. On the contrary, aversion is convinced as “a stimulus that one always avoids or prevents.” This definition soon met negative comments, and it is criticized for failure to reflect the characteristics of learning reinforcement of aversive stimuli (Murphy, 2008). Aversion learning includes two processes: (1) operant aversion to learning (1a), punishment learning (1b), and active avoidance of learning; and (2) aversive Pavlovian classical conditioning. From the perspective of biological evolution, aversion is born to sustain survival (Jean-Richard-Dit-Bressel et al., 2018). In other words, aversion is an inner feeling generated by an individual to avoid the potentially harmful stimuli (toxin, instead of predator and threat) to the body. Aversion behavior might be the contrary of reward, which includes avoidance behavior (Verharen et al., 2020). *Thus, FND patients might have problems especially at aversion behaviors: they can behave properly at rewarding behavior, but they do not know how to behave at aversive situations.*

Of note, aversion should be differentiated from disgust, which is regarded as a basic emotion. Disgust and aversion are two common psychological concepts. As we discussed above, aversion derives from motivation; it is a kind of feelings opposed to reward. On the other hand, disgust is a kind of prototypical emotion, which originates from eating toxic foods and infected by pathogens and parasites (Berridge, 2018). From this perspective, the feeling of aversion is a complex emotion, which contains a series of basic emotions, such as disgust and fear (Chapman et al., 2009). However, both disgust and aversion can induce avoidance behavior.

## Dopamine Responses to Aversive Stimuli

Even though DA was suggested to be the major neurotransmitter for reward (Salamone et al., 1993), recent studies show that various disgusting or stressful events also increased DA release or metabolism in the NAc (Davidson et al., 2004), which indicates that DAergic neurons in the limbic system may be involved in the processing of aversive stimuli (Abercrombie et al., 1989; Bradberry et al., 1991; Joseph et al., 2003; Barr et al., 2009; Fadok et al., 2009). Other scholars, however, believe that aversive stimuli belong to motivation valence, which usually induces low-level DA activities (Liu et al., 2008; Roitman et al., 2008). In addition, others suggested that this kind of DA activation is related to the processing of stimulus arousal attributes (i.e., alertness) rather than value (aversion). Further studies show that two mixed activation modes of DAergic neurons exist in the limbic system (Ventura et al., 2001).

They are coexisting high-level and low-level activation modes of DA release in different brain regions (Pascucci et al., 2007). In recent years, with the development of viral vector-based methods (e.g., cell type-specific and projection-specific electrophysiology, *in vivo* imaging, and optogenetics), researchers induce aversion in mice by exposing them to chronic or acute aversive stimuli (foot electric shock, foot shock, forced swimming test, hind paw injection of formalin, social frustration stress, and fear conditioned reflex) and by intraoral injection of the solution, which leads to oral and facial reactions (Berridge, 2000; Fadok et al., 2009; Lammel et al., 2012; Tye et al., 2013; Friedman et al., 2014). Results show that DAergic neurons can be divided into multiple subpopulations, which are responsible for the processing of different stimuli information. The DAergic neurons that are involved in processing of aversive stimuli are multiple subpopulations of midbrain DA neurons (Kim et al., 2016; Groessl et al., 2018; de Jong et al., 2019). For example, Stephan Lammel’s laboratory has been dedicated to the study of DA aversion function for many years; in their recent studies, they injected viruses into the brains of mice and measured the release of DA in different ventral striatal subregions of mice with fiber photometry under different experimental task conditions (aversion and reward conditioned reflex experiment, real-time place preference experiment, open field experiment, and approach-avoidance experiment. It is found that NAc DAergic neuron terminals in the ventral NAc medial shell (vNAcMed) was excited at aversive cues (de Jong et al., 2019). This result indicates that although most DAergic neurons in the NAc are excited by reward stimuli and reward cues, there is a cluster of DAergic neurons in the NAc that are excited at aversive stimuli and cues (Lammel et al., 2011, 2012, 2015; Yang et al., 2018; Cerniauskas et al., 2019; de Jong et al., 2019). Further studies show that during rewarding events and when aversion to toxic events decrease, DA activity in the dorsomedial NAc shell (dmNAc) increases. In contrast, the release of DA in the ventromedial NAc shell (vmNAc) was increased by both rewarding and aversive stimuli, while the DA-sensor signal in the central vmNAc (ceNAc) and ventrolateral NAc shell (vlatNAc) showed complex dynamics (Yuan et al., 2019). The same result was also found in other projection terminals of VTA DA and SN DA including the medial prefrontal cortex (mPFC) (Kim et al., 2016; Ellwood et al., 2017; Morales and Margolis, 2017; Vander Weele et al., 2018), amygdala (Fadok et al., 2009; Lutas et al., 2019), and dorsolateral and caudal striata (Lerner et al., 2015; Menegas et al., 2015). In addition to the traditional DAergic neuron aggregation areas such as the VTA and SN, dorsal raphe nucleus (DRN), and midbrain periaqueductal gray matter (PAG) have recently been found to have relatively sparse DA neuronal activities at aversive stimulation (Cardozo Pinto et al., 2019); however, there are really some DAergic neurons in these regions, which have been reported to be activated in response to aversive stimuli (Matthews et al., 2016; Groessl et al., 2018; Verharen et al., 2020). Therefore, the above research shows that some special subtypes of DAergic neurons exist in multiple brain areas of the limbic system (such as the striatum, which includes the NAc, VTA, and SN) and show excitement when aversive stimuli and cues appear.

However, some researchers still hold opposite views on whether DAergic neurons are involved in the processing of aversive stimuli. For example, Schultz doubts about the aversive function of DAergic neurons; instead, he proposed reward prediction error (Schultz, 2010). He suggested that that majority of DAergic neurons in the limbic system showed excitement toward reward stimuli and inhibition toward aversive ones. According to Schultz, DAergic neurons are excited by the learning process about both rewarding and aversive stimuli, while they make no response to aversion itself. In some studies, however, the response of DAergic neurons in the limbic system to aversive stimuli is different studies with microdialysis, rapid scanning cyclic voltammetry, and electrophysiological methods (Roitman et al., 2008; Badrinarayan et al., 2012). Based on these conflict results, some researchers proposed other assumptions on the role of DAergic neurons in the limbic system in the processing of aversive stimuli (Verharen et al., 2020). These assumptions are as follows: (1) the aversive stimulus may reflect the physical attributes rather than aversion itself (Lammel et al., 2014; Menegas et al., 2018); (2) animals experience rewards or relief when aversive stimuli terminate (Brodsky and Lajoie, 2013); (3) a high-return environment may lead to excitement toward aversive stimuli (Matthews et al., 2016); and (4) DAergic neurons transmit safety signals when an animal successfully avoids nasty events (Luo et al., 2018). These four assumptions are indeed the symptoms of FND patients; however, further studies are needed to address these problems and to provide further evidence for the involvement of DAergic neurons in the limbic system in the processing of aversive stimuli, especially in FND patients.

## Dopamine Receptors

The psychological effects of DA are mainly mediated by DA receptors, which are composed of five different but closely related G protein-coupled receptors, including D1, D2, D3, D4, and D5 DA receptors (Beaulieu and Gainetdinov, 2011). At first, Spano et al. (1978) found that only a subset of DA receptors are positively correlated with the activity of adenylyl cyclase (AC). Then DA receptors can be separated into D1-like (D1 and D5) and D2-like (D2, D3, and D4) receptors (Vallone et al., 2000; Beaulieu and Gainetdinov, 2011) based on the structure, pharmacology, biochemical characteristics, and downstream signaling pathways. D1-like receptors can enhance the activity of AC by activating Gα_s/olf_ family and can promote the production of cAMP (response element-binding protein). However, D2-like receptors activate Gs/ol family and inhibit the activity of AC and the production of cAMP (Xia et al., 2019). The five DA receptors are different in distribution and function. D1 receptors (D1Rs) are expressed mainly in the SNc; in midbrain limbic and middle cerebral cortex areas, such as the caudate–putamen (striatum), NAc, SN, olfactory bulb, amygdala, and frontal cortex; and at lower levels in the hippocampus, cerebellum, thalamic areas, and hypothalamic areas (Beaulieu and Gainetdinov, 2011). D1Rs can mediate PFC and brain-derived neurotrophic factor (BDNF) to impact partial working memory and learning process (Sarinana et al., 2014). The activation of hippocampal dentate gyrus D1Rs promotes the representation of explicit contexts and closely correlated to semantic memory (Fan et al., 2010). Besides, D1Rs also play a vital role in locomotor activity, rewarding reinforcement, and motivation. D2 receptors (D2Rs) are mainly distributed in the striatum, NAc septi, olfactory tubercle, SN, VTA, hypothalamus, cortical areas, septum, amygdala, and hippocampus (Gerfen, 2000; Vallone et al., 2000; Seeman, 2006). D2Rs play an important role in controlling motor action (Fan et al., 2010). The activation of presynaptic D2-like receptors may reduce the release of DA and then cause the reduction of motor action, but the activation of postsynaptic receptors will promote locomotor activity. The different functions of presynaptic and postsynaptic D2-like receptors may be attributed to different subtypes. It should be noted that the splice variants of the D2 DA receptors, D2RL (D2R long) and D2RS (D2R short), seem to have different neuronal distributions, with D2RS being predominantly presynaptic and D2RL being postsynaptic (Usiello et al., 2000; Mei et al., 2009). Like D1Rs, D2Rs are also vital to motivation, but they have totally different functions. It is found that there are two types of neurons projected in the dorsal striatum with two types of DA receptors, with the first type representing D1Rs and projecting in direct pathway in the basal ganglion to promote body motion, which is called rewarding approach, and the second type represents D2Rs and projects in an indirect pathway to suppress the body motion, called aversion avoidance (Hikida et al., 2010; Kravitz et al., 2010). And Kravitz et al., 2010, 2012 used ontogenetic stimuli and found that D1-medium spiny neurons (D1-MSNs) selectively activate the D1-type MSNs in the dorsal striatum, promote locomotion, and induce persistent reinforcement. In contrast, activation of the D2-type MSNs suppressed locomotion and induced transient punishment (Kravitz et al., 2010, 2012). Thus, rewarding and aversive stimuli can activate different DA neurons with different receptors and lead to the approaching or avoiding behaviors. This indicates that the rewarding and aversive neurons can be separated by different receptors. However, Al-Hasani et al. (2015) find that the activation of DAergic neurons in the ventral part of the NAc is mainly overlapped with D1-MSNs but reacts to aversive stimuli. Therefore, it is worthy further studying the different types of DAergic neurons with different DA receptors.

Although considerable progress has been made in the physiological function of D3, D4, and D5 receptors, the specific physiological functions of these receptors are yet unknown (Beaulieu and Gainetdinov, 2011), especially the function of DA neurons in rewarding and aversion. D3, D4, and D5 receptors are mainly expressed in the limbic system (Leriche et al., 2006; Rondou et al., 2010; Beaulieu and Gainetdinov, 2011). It is proved that D3 is vital to cocaine addiction (Zhang et al., 2017) and locomotion activity (Sibley, 1999). And the increase of D3 receptors (D3Rs) is closely related to tardive dyskinesia (TD) in a non-human primate model (Mahmoudi et al., 2014). Recent studies have shown that D4 receptors have the potential to improve mental disorders and cognitive functions (Guo et al., 2017). D5 receptor has beneficial effects on reward and novelty and on improving cognitive impairment (Leriche et al., 2006; Beaulieu et al., 2007; Mei et al., 2009; Shen et al., 2016). DA receptors also play a vital role in the treatment of many mental diseases, especially the FND, are targets of some psychiatric pharmaceuticals, and the target of many medications that are the main methods used to treat PD, restless legs syndrome, schizophrenia, bipolar disorder, and major depressive disorder, such as bromocriptine, pramipexole, ropinirole, chlorpromazine, risperidone, metoclopramide, brexpiprazole, and cariprazine (Pajonk, 2004; Bonuccelli et al., 2009; Fawcett et al., 2016; Kim et al., 2016; Klein et al., 2019).

## Prediction Errors and Functional Neurological Disorder

The concept of reward prediction error proposed by Schultz et al. (1997) is regarded as a milestone in the study of DA function. Along with his colleagues, Schultz found that there is a significant relationship between the activation level of VTA DAergic neurons and the reward prediction before the appearance of stimuli. Later on, Gu et al. (2019b) deduced the famous reward prediction error formula through modeling:


DAergicneuronspredictingresponse=Rewardoccurred-Rewardpredicted,

which quantifies the relationship between DAergic neuron excitement and reward prediction error. That is to say, the joy at reward stimuli is determined not only by the amount of reward but also by individual’s expectation of the reward stimuli (Wang F. et al., 2020). Chances are that the joy of a fully expected big reward is less than that of an unexpected small reward. Correspondingly, DAergic neurons perform differently at expected errors (as expected, better than expected, and worse than expected). Rewards regarded as “expected” might incur tonic DA release, those as “better than expected” produce phase burst signals, and those as “worse than expected” generate suspension of activation of DAergic neurons. Bursts of action potentials induce more DA release in a specific projection area than spikes of the same number in the spaced action potential tissue (Pignatelli and Bonci, 2015). It is worth noting that DAergic neurons in the limbic system are traditionally believed not to be sensitive to aversion prediction errors, because the midbrain DAergic neurons might only process the information of reward prediction errors, while they make no response in accordance with the occurrence of aversive clues [conditioned stimuli (CSs)] (Fiorillo et al., 2013; McHugh et al., 2014).

However, a recent study showed a totally different result, in contrast to previous studies that showed that the VTA and SN in the traditional DA neuron aggregation area are not activated by aversion prediction errors (Mileykovskiy and Morales, 2011; Schultz, 2015), and even showed inhibitory response to aversion prediction cues (Ungless, 2004; Tan et al., 2012). However, related results show that midbrain DAergic neurons are distributed not only in the VTA and SN but also in the PAG and DRN (Dougalis et al., 2012). Despite sparse distribution in the VTA and SN, these DAergic neurons are highly likely to play an important role in aversion prediction errors according to the characteristics of neurons in these brain areas (Matthews et al., 2016; Cardozo Pinto et al., 2019). Groessl et al. (2018) investigated the activities of DAergic neurons at CSs (such as noise) and unconditioned stimuli (USs; such as foot shock). Results showed that PAG and DRN DAergic neurons are activated by the aversive stimuli clues (noise). These mice showed three kinds of prediction errors: as expected, worse than expected, and better than expected. It turns out that the DAergic neurons of the PAG and DRN produced a potential activity similar to that of the DAergic neurons of the VTA during the reward prediction error. In other words, aversion regarded as “as expected” will incur tonic release; reward as “worse than expected” will incur a phase burst signal; and aversion as “as expected” will incur a suspension of activation. These results suggest that the DAergic neurons of the PAG and DRN are likely to be involved in the processing of aversive prediction errors.

Besides, human beings might also follow this principle in the process of decision making. Nobel Prize winner Professor Kahneman proposed in his classic loss aversion theory that human beings also follow the principle of seeking advantages and avoiding disadvantages in the process of decision making. Decision making under risk usually expresses different levels of aversion preferences and shows higher sensitivity to potential aversion than the same level of acquisition (Kahneman and Tversky, 1979). The involvement of the PAG in the processing of aversive prediction errors has been demonstrated in a functional magnetic resonance imaging (fMRI) study on the human brain (Roy et al., 2014). Roy et al. (2014) compared two different tests on aversion to avoid acquisition and degree of pain, and then they established two types of prediction errors. One is “all or nothing” and the other “continuous change.” Changes in relevant brain regions under three prediction errors (as expected, worse than expected, and better than expected) are recorded using fMRI. With Bayesian statistics processing, the results showed that the PAG is the key brain region of prediction error processing; at the same time, an important loop of aversive prediction error is identified: PAG–mPFC. Due to the intrusive nature of neuronal imaging (Verharen et al., 2020), it is not yet determined whether it is DAergic neurons that are activated by aversive prediction errors in the PAG. But with research results in mice by Groessl et al. (2018), it can be speculated that there exist a cluster of DAergic neurons in the PAG of animals that respond to aversive stimuli and engage in the processing of aversive prediction errors. The activation of aversive prediction errors in the human brain is likely to be incurred by a subgroup located in the area of DAergic neurons.

According to the description of the *Diagnostic and Statistical Manual of Mental Disorders*, fifth edition (DSM-5), FND is defined as a neurological disease with symptoms (such as motor and cognitive disorder) that cannot be explained by current neurological theories. When it comes to behavior research, FND patients are different in emotion and recognition from normal people, embodied by their more negative emotion and worse prediction (Pick et al., 2019). An fMRI study of FND patients found that the PAG activation increases when they face facial expressions, and the connection of the dorsolateral PFC (dlPFC) and insula increases according to an emotion-related study on them (Aybek et al., 2015; Espay et al., 2018; Szaflarski et al., 2018). Therefore, it is fair to say that the behavior and fMRI results are both strongly correlated with DAergic neurons in the PAG area. Accordingly, it is predicted that the abnormality of DAergic neurons in the PAG area is one of potential causes of FND. FND patients often show hysterical behaviors in stressful situations. FND patients tend to be more negative at loss and gain lower feelings of happiness from reward than healthy people. Consequently, they feel exhausted, indifferent, and even depressed, which appears to be consistent with symptoms of lacking DA (Logan and McClung, 2019; Xia et al., 2019; Liang et al., 2021). In conclusion, it is necessary to further explore the activation difference of DAergic neurons between FND patients and healthy people with predicting reward or aversion, so as to uncover the causes for FND.

## Parkinson’s Disease and Functional Neurological Disorder

There are symptoms of movement disorders at the onset stage of both PD and FND, for example, tremors; and related studies have found that the activation patterns of the basal ganglia are abnormal in patients with both diseases (Lehn et al., 2016; Klein et al., 2019). One of the main input areas of the basal ganglia is the striatum. Ninety percent of neurons in the striatum are GABAergic spiny projection neurons (SPNs), which highly express D1Rs and D2Rs and control movement by DAergic projections (Gerfen and Surmeier, 2011). Nowadays, it has proved that PD is related to the abnormalities in the pathway (Bonuccelli et al., 2009; McKinley et al., 2019), and recent studies of FND have also found that motor conversion disorders in FND patients may be related to abnormalities in this area (Voon et al., 2010; Lehn et al., 2016). PD is a common neurodegenerative movement disorder (Klein et al., 2019), which can be mainly divided into two subtypes. The first type is tremor-dominant PD whose symptoms are mainly motor retardation, muscle rigidity, resting tremor, and posture and gait disorder. The second type is non-tremor-dominant PD, which usually progresses faster and has higher functional disability than tremor-dominant PD (Jankovic et al., 1990; Kalia and Lang, 2015). The common incidence of PD happens in the elderly over 60 years old, and the incidence rate is about 13 cases per 100,000. And the incidence rate of men is higher than that of women (Williams-Gray and Worth, 2016).

The main motor symptoms of PD are caused by the degeneration of SN DAergic neurons (Suárez et al., 2014), which mainly project in the dorsal striatum. The main function of the striatum is to evaluate the “action plans” produced by the cerebral cortex; to construct a movement diagram based on the sensory state, motivational state, and past experience; and then to transmit the evaluation to other basal ganglion nuclei (Zhai et al., 2019). At the receptor level, the abnormal regulation of the direct and indirect pathway spiny projection D1Rs and D2Rs in the dorsal stratum is the cause of the main motor symptoms of PD (Gerfen and Surmeier, 2011). It was suggested that D1Rs and D2Rs control the “direct pathway” and “indirect pathway” and promote or inhibit body movements, respectively (Xia et al., 2019). And it has been found that the excitability of D1 and D2 shifted in opposite directions in PD patients, causing an imbalance in the regulation of the thalamus movement (Gerfen and Surmeier, 2011). Meanwhile, some studies have shown that the lack of DA alters the induction of long-term plasticity at glutamatergic synapses, and the lack of D1R signal may cause the long-term bias of the direct pathway of glutamatergic synapses, but the lack of D2Rs signal can cause long-term potentiation (LTP) of direct pathway glutamatergic synapses (Mallet et al., 2006). Thus, the current treatment for PD is using drugs that increase DA concentration or directly stimulate DA receptors for symptomatic treatment.

Similarly, FND symptoms are characterized by physical symptoms without disease pathology, mainly manifested as tremor, myodystonia, and gait disorder (Voon et al., 2010). FND patients account for about 30% of neurological outpatients (Carson et al., 2003). FND patients also suffer more than other known mental illness patients, because the causes of FND are not yet clear. At the same time, severe FND can also cause physical disability (Pick et al., 2019). In addition to physical symptoms, FND patients often report a series of emotional dysfunctions, such as anxiety, depression, alexithymia, and/or affective regulation disorder (Brown and Reuber, 2016; Carson and Lehn, 2016). Recently, some researchers have begun to study the differences between FND patients and normal people from the perspective of emotional processing and put forward some hypotheses for the causes of FND from the perspective of emotion. For example, Voon et al. (2010) applied an fMRI in studying the difference in brain activation between normal subjects and FND patients in recognizing emotional faces. They found that there was no difference of the activation of the amygdala when normal people watch emotional faces (happiness, neutral, and fear). And further analysis has found a greater interaction between the right amygdala and the right supplementary motor area in FND patients compared with normal subjects (Voon et al., 2010), which indicates some abnormalities in the amygdala of FND patients. The basolateral amygdala receives the signals from the hippocampus and cerebral cortex and then projects to the dorsal and ventral striata. Meanwhile, the basolateral amygdala can also send projections to the central nucleus of the amygdala and the terminal striae of the lateral basilar nucleus (extended to the amygdala) and then project to the gray matter around the aqueduct, the LHa (autonomous responses), and the midbrain nucleus, such as the VTA (Lang and Davis, 2006). The DAergic neurons projected via both pathways are related to motor control (Zhai et al., 2019) and reward prediction errors (Schultz et al., 1997; Groessl et al., 2018). The greater functional connectivity of the marginal zone in FND patients affects the motor preparation zone, which may be a pathophysiological basis of FND (Voon et al., 2010). Schrag et al. (2013) have some similar findings. Anyway, the causes of FND are complicated, and FND may not be caused by a dysfunction of a single brain area. Therefore, future studies are needed to start with the interactions between emotion and motor brain area and to study the DAergic projection in different neural networks.

## Neural Networks for Dopamine Projection

The limbic system has been called the emotional brain, which is located at the underside of the brain and the inner side of the brain [including the anterior cingulate cortex (ACC)], the lower corpus callosum, the hippocampal structures in the hippocampus and deep hippocampus, and some subcortical brain structures, including the thalami, VTA, and SN (Shen and Lin, 1993; He et al., 2009). The VTA and SN in the ventral side of the limbic system have long been considered as the main source of DA transmitters in the cerebral cortex and subcutaneous tissue (Bjorklund and Dunnett, 2007). This area is also involved the processing of reward prediction error calculation (Schultz et al., 1997), with gamma-aminobutyric acid (GABA) in the VTA regulating the amount of reward by inhibiting the activity of DAergic neurons (Roberts et al., 2021), thereby calculating the reward prediction error (Cohen et al., 2012). At the same time, some animal studies have indicated that there are multiple clusters of neurons in this region that are responsible for different motivational functions and may be involved in different neural circuits to process reward and aversive information (Lammel et al., 2011). However, most studies mainly focus on the reward function of DA in the limbic system, lacking studies on the aversion function and its neural circuits. In addition, some researchers even questioned the aversive function of DA neurons in the limbic system (Schultz, 2010).

Bromberg-Martin et al. (2010) proposed that DAergic neurons of the limbic system act on different motivational controls when they participate in different neural networks. Therefore, it has been a vital research direction in the related field to find the aversive circuit of DAergic neurons of the limbic system. In recent years, with the development of virus-tracking technology, some aversive circuits of DA have been discovered (Hu, 2016; Verharen et al., 2020). For example, some studies have reported four limbic system brain areas: the VTA, SN, PAG, and DRN. The next discussion about the aversive circuit of DAergic neurons in the limbic system will also focus on these brain areas.

## The Aversion Function of Dopaminergic Neurons in the Ventral Tegmental Area

**FIGURE 1:**
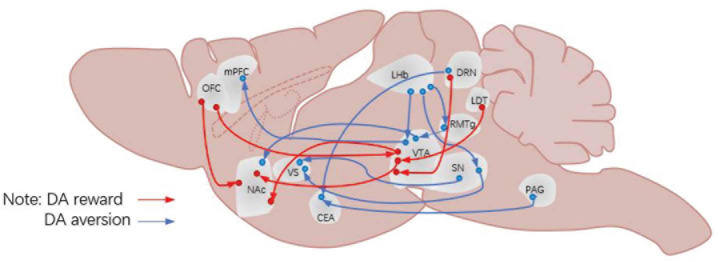
Neural circuits for DA projection. DA, dopamine; OFC, orbitofrontal cortex; mPFC, medial prefrontal cortex; NAc, nucleus accumbens; LHb, lateral habenula; VS, ventral striatum; CEA, central amygdala; VTA, ventral tegmental area; SN, substantia nigra pars compacta; RMTg, rostromedial tegmental nucleus; DRN, dorsal raphe nucleus; PAG, periaqueductal gray matter; LDT, laterodorsal tegmental nucleus.

The VTA is an important nucleus for DAergic neurons, which play an important role in reward prediction error. Indeed, the mesolimbic DA system (refer to the DA pathway from VTA DAergic neurons to the NAc) has always been considered to play an important role in processing information about rewards, approaching behaviors and positive reinforcement (Saunders et al., 2018; Yuan et al., 2019). However, some researchers suggested that the DA release of neural circuits connected to DAergic neurons in this region is involved in the processing of aversive stimuli. For example, Lammel et al. (2011, 2012) discovered a circuit of VTA DAergic neurons processing aversive stimuli. Many new ways of studying have shed some light in this field, by injecting viruses and fluorescent tracers into the VTA of mice; applying conditioned place preference (CPP), conditioned place aversion (CPA), and open field tasks to these mice; and recording the activities of DAergic neurons in the VTA. And these results showed that there are two kinds of VTA DAergic pathways: the rostromedial tegmental nucleus (RMTg) → VTA DA → dorsal NAc pathway, which is mainly responsible for processing rewarding stimuli, and the other one is the LHb → VTA DA → mPFC pathway, which is involved in processing the aversive stimuli (Figure 1). Besides, there are studies that further reported several subgroups of DAergic neurons in the dorsal VTA, which are embedded in different circuits and participated in different behavioral functions. However, these pathways have been questioned by some later studies (Tian and Uchida, 2015; Tian et al., 2016); for example, Tian et al. (2016) showed that aversive stimulus processing function was not affected by VTA impairment, which suggested that the reaction is insensitive to the decline of the frequency of rewards. However, this aversive pathway is supported by many other studies (Pignatelli and Bonci, 2015; Hu, 2016). Alternatively, de Jong et al. (2019) reported another pathway for aversive processing associated with VTA DAergic neurons, which is LHb → RMTg → VTA DA → vNAcMed (Lerner et al., 2015). In all, these studies indicate that some DAergic neurons participate in the processing of aversive stimuli through the VTA as a start point projecting to different brain regions.

## The Aversive Function of Dopaminergic Neurons in the Substantia Nigra

Like the VTA, the SN is also an important nucleus of DAergic neurons. Some recent studies also found some aversive pathways related to SN DAergic neurons. Matsumoto and Hikosaka (2009) conducted a study about Pavlov’s classical conditioned reflex and found that the LHb → SN DA → dorsal striatum (dVS) pathway is likely responsible for processing aversive stimulus. And the same result was also found in the SN DA → ventral striatum (VS) pathway. Menegas et al. (2017, 2018) found that the SN DA → VS pathway played an important role in the fear aversion learning task of rats. The discovery of the pathway is consistent with their previous findings that lateral SN DAergic neurons are involved in the processing of aversive stimuli.

## Aversive Function of Periaqueductal Gray Matter and Dorsal Raphe Nucleus Dopaminergic Neurons

Unlike the VTA and SN, the presence of DAergic neurons in the PAG and DRN has only been discovered in recent studies (Cardozo Pinto et al., 2019). Therefore, there is still some controversy about whether there are DAergic neurons in these two brain regions. With the advent of electrophysiology and other biological imaging techniques, some researchers have begun to study the characteristics of DAergic neurons in these two regions (Dougalis et al., 2012; Li et al., 2016) and have made a series of reports, which further support the existence of DAergic neurons in these two brain regions. Similarly, some studies have reported about the aversive function of PAG and DRN DAergic neurons, which have shown that the two brain regions (the PAG and DRN) play an important role in coping with pain stimuli (Eippert and Tracey, 2014; Gadotti et al., 2019), and the amygdala is an important targeting area of these two brain regions (McNally and Cole, 2006). Therefore, it is important to explore whether the PAG/DRN → amygdala circuit is involved in the processing of aversion stimuli. The study conducted by Groessl et al. (2018) found that PAG and DRN DAergic neurons play an important role in the processing of aversive stimulus and aversive prediction error processing (Charney, 2004). At the same time, it is found that PAG and DRN DAergic neurons projected to the central amygdala (CE) and confirmed that the PAG/DRN → CE circle is responsible for the aversive stimulus and the processing of aversive clues.

In addition to the above regions, DAergic neurons in other areas in the limbic system have also been found to be engaged in the processing of aversive stimuli. A recent study of Kramar et al. (2021) showed that the VTA DA release promotes the activation of DAergic neurons in the dorsal hippocampus, which reinforces late consolidation of aversive memory (Clewett and Murty, 2019; Shao et al., 2021). The hypothalamus is an important area of emotional processing in the limbic system with its medial and lateral nuclei processing different information. The medial nucleus is mainly responsible for the processing of reward stimuli such as food intake and sexual behavior, while the lateral nuclei are responsible for the processing of aversive information including stress and tension (Barbosa et al., 2017). It is worth noting that a recent study found that LHb projection to LHA neurons is responsible for encoding aversive information (Lazaridis et al., 2019). As Lammel et al. (2012) previously reported that aversive information is processed by DA transmission of (LHb) → VTA DA → mPFC circle, is the LHb → LHA projection related to DAergic neurons? Therefore, further research should focus not only on traditional distribution of DAergic neurons mentioned above but also on whether other areas of the limbic system and DAergic neurons in nuclei are involved in the processing of aversive information.

## Dopamine and Norepinephrine

**FIGURE 2:**
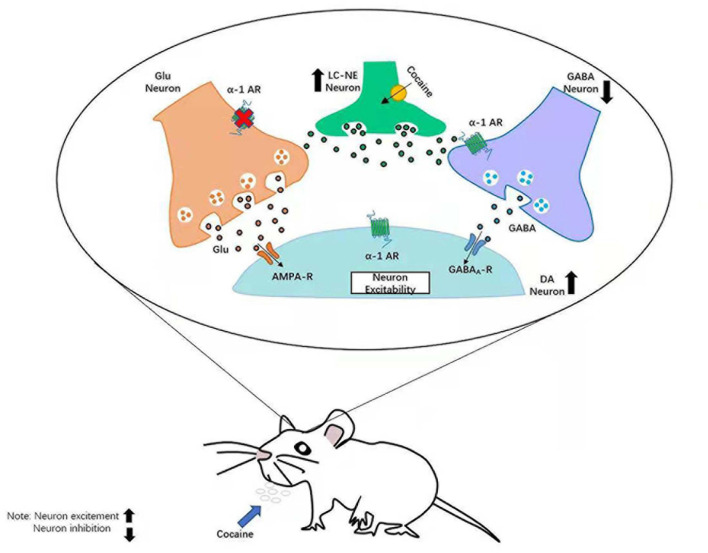
Neural mechanism of LC–NE affecting DA in addictive behavior. LC, locus coeruleus; NE, norepinephrine; DA, dopamine.

Dopamine and norepinephrine (NE) neurons project widely in the whole brain (Charney, 2004). NE is mainly produced by neurons in the locus coeruleus (LC), and the LC–NE systems respond to stress by globally priming neurons in the brain (Nestler and Aghajanian, 1997; McCall et al., 2015). The already known functions of NE are alerting (Mandalaywala et al., 2017), arousal (Carter et al., 2010), recognition (Wagatsuma et al., 2018), sleep/awake transitions (Carter et al., 2012), and drug addiction (Cao et al., 2010). Some recent studies have also found that the LC–NE is related to stress recovery (Valentino and Van Bockstaele, 2015; Liang et al., 2021; Sullivan and Ballantyne, 2021) as the function of the pursuit of reward and the avoidance of punishment in DA and the function of alerting and arousal in norepinephrine are respectively relative to the valence and arousal in the theory of emotional dimension. Wang F. et al. (2020) added serotonin (5-HT) as another neurotransmitter and put up “three primary color model” of basic emotions (Gu et al., 2019b). The theory proposed that all emotions are composed of some basic emotions, such as happiness, sadness, and anger and fear, which are subsided respectively by the three neurotransmitters: DA, happiness; 5-HT, sadness; and NE, fear (anger). Recent studies have indicated that the VTA is a downstream projection area of the LC (Isingrini et al., 2016), and the LC–NE neurons manage negative emotion through suppressing VTA DAergic neurons. Current studies on the effect of NE transmitter to DAergic neurons focusing on addictive behavior (Grace et al., 2007) and the treatment of depression (Friedman et al., 2016; Gu et al., 2020) suggested that the LC–NE neurons projection to midbrain DA neurons and that their target structures play a vital role in the changes of neural circuits. These changes might be the causes for pathological behaviors, including the development and maintenance of addiction and the alteration of the reward and aversion neural circuits in patients with addiction or depression (Everitt and Robbins, 2005; Hyman et al., 2006; Schultz, 2007; Admon et al., 2017). It is crucially important to explore the mechanisms of changes of DAergic neurons, which are modulated by NE neurotransmitters. Relevant studies have shown that DAergic neurons are tensely distributed with α1 and β3 adrenergic receptors (Zhang et al., 2019). In the process of reward, the VTA DAergic neurons mainly receive the projection of glutamic acid (Mingote et al., 2019; Wang J. et al., 2020), GABA (Bouarab et al., 2019), and NE neurons. And the main function of DAergic neurons is to suppress the activation of GABAergic neuron. On the one hand, α1-Ars receptors can promote release of DA from DAergic neurons (Mitrano et al., 2012). In addition, α1-Ars receptors can also affect the release of GABA directly in the VTA DAergic neurons (Velasquez-Martinez et al., 2015) and further strengthen the activation of DAergic neurons (Figure 2). Velasquez-Martinez et al. (2015) found that cocaine-induced inhibition of monoamine reuptake may further enhance the availability of NE in synapse and invoke stronger stimulation of α1-ARs but may reduce the release of GABA in the VTA neurons (Martins et al., 2019). It may reverse neurotransmitter actions related to aversive behaviors, increase the nervous excitability of DAergic neurons, and then cause sensitization to drugs like cocaine (Clewett and Murty, 2019; Velasquez-Martinez et al., 2020). Zhang et al. (2019) reported in a study of social pressure and sensitivity that mice with stronger psychological resilience will release more NE than those with weaker psychological resilience in the circumstance of depression. After depressive-like behaviors were reversed, NE release increased rapidly, which indicates that the LC–NE system has an important function in mediating the psychological resilience of humans and animals.

In summary, NE (especially α1-Ars) participates in the processing of reward information of the VTA DAergic neurons, which directly promote the activation of DAergic neurons and hinder the release of antagonist GABA of DA. It will further affect the processing of reward information to the VTA DA and is significantly meaningful to the relevant treatment of addictive behavior, depression, and some diseases. Meanwhile, other studies (Mitrano et al., 2012) have found that the function of DA system and NE system interacts in other brain regions, which affect many psychological processes.

## Dopamine and Serotonin

5-HT was first suggested as a neurotransmitter for aversion in Bradley et al. (1986), and there is a great overlap in function of both 5-HT and DA (Yagishita, 2020). But 5-HT plays a vital role in motivation-related functions, such as reward and aversion learning, incentive processing, decision making, and goal-oriented behavior (Hu, 2016). The main gathering area for 5-HT neurons is the DRNs. The 5-HT neurons of the DRN send projection to the entire brain, including the striatum and thalamus (including the posterior complex and the lateral geniculate nucleus, the anterior ventral nucleus, and the anterior ventral nucleus), cingulate cortex, PFC (including medial PFC), temporal lobe, and sensory cortex (Conio et al., 2020), and receive the input from the hypothalamus, amygdala, midbrain, and the anterior neocortex (Weissbourd et al., 2014). The types of 5-HT receptors are more complicated than those of DA. There are 14 types of 5-HT receptors and can be divided into seven main families according to different G-proteins coupled. Different receptors in different families have different functions. For example, 5-HT1 plays a role in rewards (Akizawa et al., 2019) and anxiolytics (Ramboz et al., 1998), 5-HT2A receptors are correlated to hallucinations and insights (Vollenweider et al., 1998); 5-HT4 receptors have an effect on memory, depression, and feeding (Bockaert et al., 2008); and 5-HT7 is related to cognitive processes, such as learning and memory (Zareifopoulos and Papatheodoropoulos, 2016).

Although the psychiatric DA hypothesis has been the main hypothesis in the field of psychiatry in the past 50s, almost all drugs for the treatment of psychosis involve DA receptors, especially D2Rs (Stahl, 2018). However, more and more studies have found that 5-HT is also vital to treating mental disorders. Some symptoms of mental patients can also be alleviated by drugs interfered with 5-HT or 5-HT receptors in related brain regions (Stahl, 2016). Therefore, some scholars put forward that psychosis is a condition involving the disorder of multiple neurotransmitters in multiple pathways, but DA and 5-HT may be functioned in some psychosis processes at the same time (Rolland et al., 2014). New therapies for pathways and receptors besides D2Rs in the mesolimbic pathway have received more and more attention. The classic psychiatric DA hypothesis indicates that the abnormal DA pathway in the middle-marginal DA pathway is the cause of most psychosis, and almost all antipsychotic drugs block D2Rs there (Stahl, 2018).

However, a large number of clinical researches have indicated that blocking the D2Rs in this pathway will simultaneously block the D2Rs in the pathway of the SN to the dorsal striatum, leading to some motor disorders, such as meditation, drug-induced PD, and long-term TD (Stahl, 2016). Meanwhile, although the method of blocking D2Rs is effective for patients with manic, depressive psychosis and schizophrenia, it is not ideal for treating PD, Alzheimer’s, and other types of psychosis and even makes the symptoms worse (Ravina et al., 2007; Fénelon et al., 2010). Therefore, researchers have started to study other neurotransmitters in the treatment for psychiatric patients. The 5-HT theory is proposed in this context, which believes that psychosis may be the result of excessive activation of 5-HT2A receptors in glutamate neurons. Thus, blocking the overdose neurotransmission of 5-HT in patients with 5-HT2A receptors in psychosis can theoretically restore the balance between 5-HT and DA and can reduce visual hallucinations and delusions instead of worsening motor symptoms. It can effectively treat PD and Alzheimer’s (Stahl, 2018). Current studies have shown that the loss of DA in the dorsal striatum due to the SNc in patients of PD will change the normal balance between 5-HT and DA. Meanwhile, the 5-HT neurons in the fissure will degenerate with the original loss of 5-HT and worsen with the PD. Pyramidal neurons in 5-HT-related brain regions degenerate, and the number of 5-HT2A receptors is increased in the remaining neurons in the cerebral cortex, which leads to hallucinations in patients (Ballanger et al., 2010; Huot et al., 2010). In other words, 5-HT abnormalities are not significantly related to motor symptoms in PD but may be related to non-motor symptoms. Related clinical studies have found that 5-HT2A antagonists without d2 antagonists have been shown to be effective in the treatment of psychotic symptoms caused by PD. Among the second-generation antipsychotic drugs, 5-HT2A receptors are also important targets for drug theory. Simultaneous targeted therapy of DA D2Rs and 5-HT 5-HT2A receptors shows better performance than a single intervention on D2Rs and fewer side effects (Xia et al., 2019). To sum up, FND patients show obvious physical symptoms and mood disorders. 5-HT is related to many affective and executive functions. Related researches have indicated that 5-HT is associated with anxiety (Deakin and Graeff, 1991), depression (Kraus et al., 2017), alexithymia (Li et al., 2020), and emotion regulation disorder (Stiedl et al., 2009). Meanwhile, the function of 5-HT and FND patients in rewarding and aversion processing has received more and more attention. The aversive function of 5-HT has been early discovered (Bocchio et al., 2016), and behavior inhabitation (Crockett et al., 2009) has also been proved to be a vital function of 5-HT. And it is found that the NAc, meanwhile, receives the projection of 5-HT, and these neurons projected by 5-HT are also activated when receiving the projection of rewarding stimuli, which shows that it is closely related to rewarding (Barot et al., 2007). And DRN 5-HT neurons are excited about the actual reward (Liu et al., 2014) regardless of whether the reward is predicted or not. Therefore, considering that 5-HT is involved in the two major obstacles of FND at the same time, drug intervention in the 5-HT concentration of FND patients or drug treatment with 5-HT receptors as a target may alleviate some symptoms of FND patients to a certain extent. In the future, it is necessary to further clarify the role of DA and 5-HT in the common projection area (such as the NAc) domain, which is meaningful to intervene related transmitter receptors precisely and cue some related mental diseases.

## Shortcomings and Prospects

The aversive function of DAergic neurons in the limbic system has been confirmed by many experiments, and the results have gradually obtained unanimous agreement. Many regions in the limbic system such as the VTA, SN, PAG, DRN, and VS (NAc) are suggested to be involved in processing aversive stimuli, and more relevant studies are gathering momentum. However, there are still some concerns in this area of research.

First, the technology of DA measurement is of concern. Problems still exist despite that significant progress has been done in the DA measurement with improved technology of electrophysiology, imaging, and virus tracking; DA imaging is still impossible to be applied to the human brain due to its invasive nature (Verharen et al., 2020). It can be speculated that DA in the human brain is engaged in the processing of aversive stimuli according to research on mice and rhesus monkeys. Humans, however, as higher organisms, show significant difference on brain structure and function with mice and monkeys. Further studies are needed on whether DAergic neurons in the limbic system of the human brain operate and function the same as the brains of mice and monkeys.

Second, whether DAergic neurons can be identified accurately emerges as a more important question. Morales and Margolis (2017) raised doubts about the identification of DAergic neurons in the VTA in the experiments. For example, neurons in mice can produce mRNA for tyrosine hydroxylase (TH) but cannot synthesize the enzyme required for DA. Therefore, false positives may be caused by identifying or manipulating DA neurons in the experiment on TH mRNA of mice (Yamaguchi et al., 2015).

Another concern relates to the ambiguous definition of aversion. Biologically, an aversive feeling is defined as an inner feeling when a creature avoids potential harm of stimuli (toxins, predators, and mechanical stress) on body for survival (Tovote et al., 2016). This definition, however, is too broad, as it includes almost all negative emotions and it leads to many problems in specific experiments. Stimuli in psychology and biology such as fear, pain, and social isolation are all considered as aversive stimuli; their value and degree of arousal, however, lack clear distinction (Lammel et al., 2012; Li et al., 2016; Matthews et al., 2016). So which stimulus incurs the reaction of DAergic neurons? Is it fear, pain, or the psychological effects of social isolation? Failure to distinguish value (aversion) and arousal (alertness) will lead to the confusion between aversion and alertness. Which feature of the stimulus produces a high level release of DA (Bromberg-Martin et al., 2010; Gu et al., 2019a)? Therefore, more studies are needed in studying how to further distinguish biological aversive stimuli, especially the distinction between value and arousal.

## Conclusion

To sum up, the article starts with the aversion function of DA, discussing the definition of aversion. Then according to series of studies, it discusses some special subtypes of DAergic neurons that exist in multiple brain areas of the limbic system. Though some researchers hold opposite views on whether DAergic neurons are involved in the processing of aversive stimuli, they show excitation when giving aversive stimuli and cues. Then, the article discusses the prediction error function of DA. According to some performances of FND patients in event prediction, it is speculated that the abnormal prediction error loop of DA may be the potential cause of FND. Next, the article summarizes the neural circuits that DA neurons in the limbic system participate in the processing of aversive stimuli and propose some assumptions about neural circuits of DA projection. After that, it discusses the relationship of NE and DA based on the researches of addictive behavior and the treatment of depression. Finally, shortcomings of current researches and prospects of future study are summarized, attempting to help further research on DA.

## Author Contributions

ZH, YJ, DQ, and FW conceived the study. ZH, YJ, SG, FW, JH, GF, and XM wrote and revised the manuscript. All the authors contributed to the article and approved the submitted version.

## Conflict of Interest

The authors declare that the research was conducted in the absence of any commercial or financial relationships that could be construed as a potential conflict of interest.

## Publisher’s Note

All claims expressed in this article are solely those of the authors and do not necessarily represent those of their affiliated organizations, or those of the publisher, the editors and the reviewers. Any product that may be evaluated in this article, or claim that may be made by its manufacturer, is not guaranteed or endorsed by the publisher.
